# A Fluorescence-Based Temperature-Jump Apparatus for Illustrating Protein Dynamics on the Millisecond Time Scale

**DOI:** 10.1021/acs.analchem.4c06501

**Published:** 2025-02-13

**Authors:** Liang-Che Kung, Li-Kang Chu

**Affiliations:** Department of Chemistry, National Tsing Hua University, 101, Sec. 2, Kuang-Fu Road, Hsinchu 300044, Taiwan

## Abstract

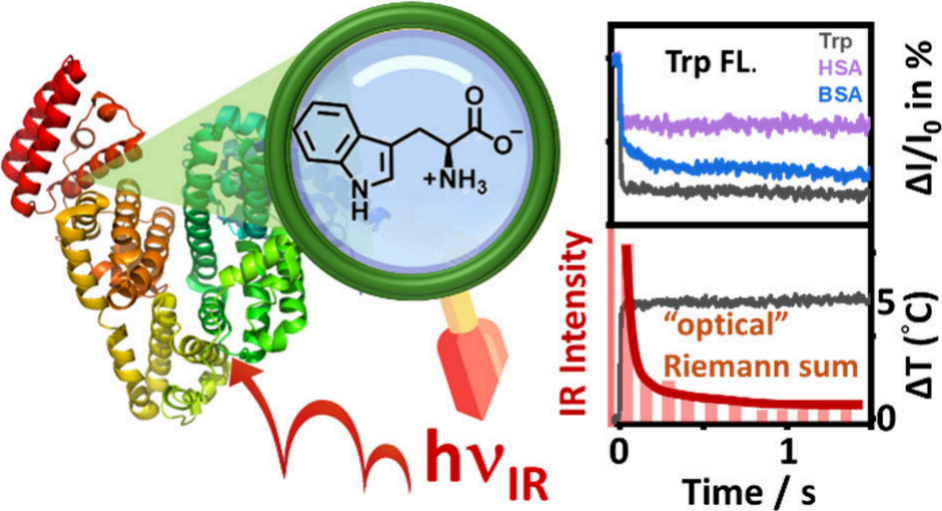

A fluorescence-based temperature jump (T-jump) module was constructed to illustrate the large-domain motion of a given protein upon thermal stimulus on the millisecond time scale. The aqueous sample was readily heated by 5.0 °C in ca. 2 ms with a lasting high temperature plateau (>1 s) upon irradiation with the “optical Riemann sum” of the discrete infrared pulses of different energy sequences from a 1467 nm diode laser operated at 1k Hz. The temperature evolution was revealed by the time-evolved fluorescence intensity change of the dissolved tryptophan. Bovine serum albumin (BSA) and human serum albumin (HSA) were chosen as model proteins, and their fluorescence intensity evolutions were recorded at 36.6–39.9 °C upon T-jump from 35.0 °C, within the range of physiological temperatures. The observed protein dynamics of BSA was characterized with an apparent activation energy of 276 ± 23 kJ mol^–1^, whereas HSA did not manifest the dynamic component. In this measurement, only a tiny amount of sample, ca. 1 μL, was required due to the conjugation of the microspot objective, and the initial temperature was readily controlled by a homemade thermostatic pad. This millisecond-resolution technique is advantageous for illustrating the large-domain dynamics of the targeted protein, bridging the characterizations of the localized protein dynamics on nanosecond to microsecond time scales using the fast techniques and the steady-state protein conformational features by conventional methods, such as Fourier-transform infrared and circular dichroism spectroscopies.

## Introduction

A protein with functions relies on its structure and the concomitant structural responsivity toward environmental perturbations and stimuli. The protein dynamics, referring to the reversible or irreversible time-dependent fluctuations of atomic coordinates,^1,2^ includes the various conformational changes for the “motions” along the energy landscape^3^ on wide time scales from subfemtoseconds to seconds.^4^ Miscellaneous experimental techniques have been used to probe the dynamic processes of a given protein.^5−31^ For example, nuclear magnetic resonance (NMR) relaxation^5,6^ and time-resolved cryo-electron microscopy^7,8^ are capable of providing (near) atomic-resolution snapshots of the transition between tier-0 substrates, which refer to the collective large-domain motions occurring in spans of microseconds to milliseconds.^4^ These “slow” dynamic processes strongly correlate to protein folding, ligand binding, enzyme catalysis, and allosteric regulation.^2,5,9^

Temperature jump (T-jump),^10−31^ coupled with various spectroscopic detection methods, such as infrared,^10,23,26^ two-dimensional infrared (2D IR),^14,19,27^ Raman,^13^ circular dichroism,^18^ and fluorescence-based detection,^12,15,17,20−22,24,25,28−31^ covers a wider time scale from picoseconds to seconds to study the site-specific, local and global relaxation dynamic processes of a given protein upon thermal stimuli. The temperature of the solution can be increased by capacitor discharge,^12,15,24,32^ photoexcitation of dyes^10^ and gold nanorods,^25^ and direct excitation of H_2_O and D_2_O at 1.5–2.0 μm by infrared photons from an optical parametric oscillator/amplifier (OPO/OPA),^13,14,18,19,21,26,28,29^ diode laser,^17,30,31^ Raman shifter using H_2_, D_2_, and CH_4_ gases,^16,20,22,23,33,34^ and continuous-wave (CW) fiber laser.^27^ Generally, an instantaneous temperature increase can be achieved within <100 ns and the high temperature plateau can last for <10 ms upon the nanosecond pulsed IR heating of water.^16^ Unfortunately, the cascading processes, occurring in milliseconds to seconds, include the thermalization of the heating volume, thus hampering the correct characterization of the protein dynamics at the jumped temperatures. Moreover, the optical alignment can be quite challenging because the beam size of the ultraviolet light/laser (∼ tens of micrometers) for exciting the protein solution to generate tryptophan fluorescence is restricted to a size smaller than that of the infrared heating beam (∼ hundred(s) of micrometers) to ensure alteration in the fluorescence intensity generated in the heating volume.^16^

In this work, we constructed a fluorescence-based T-jump system upon optical heating of water with an intensity-modulated infrared diode laser. A similar heating concept has been developed by Tokmakoff’s group, who used a sophisticated apparatus with an intense continuous-wave laser modulated by an acousto-optic modulator (AOM).^27^ In the present setup, the intensities of discrete infrared pulse sequences from a commercially available diode laser can be readily programmed to achieve a jumped temperature of ca. 5 °C in ca. 2 ms, conceptually referring to an “optical Riemann sum” heating process. Moreover, an objective was employed to probe the fluorescence signal from a tiny heating volume of ca. 1 μL. To verify the feasibility of this apparatus, the protein dynamics of HSA and BSA and the corresponding kinetics were analyzed. This millisecond-resolution technique is advantageous for studying the large-domain protein dynamics, bridging the fast techniques for investigating the local structural changes on nanosecond/microsecond time scales and the steady-state spectroscopic methods to reflect the stationary protein features.

## Experimental Section

### Chemicals

l-Tryptophan (Trp, 93659–10G, Sigma-Aldrich, ≥99.5%), human serum albumin (HSA, SI-A5843-BCCG2112–5G, Sigma-Aldrich, ≥96%), bovine serum albumin (BSA, A1933–5G, Sigma-Aldrich, ≥98%), hydrochloric acid (HCl, 30721–2.5L-GL, Honeywell, ≥37 wt %), and tris(hydroxymethyl)aminomethane (Tris base, T6791–100G, Sigma-Aldrich, ≥99.9%) were purchased for use without further purification. Deionized water with a resistivity of 18.2 MΩ cm was obtained from a water purification system (SIPK0SIA1, Merck Millipore).

### Sample Preparations

The 10 mM tris base buffer was prepared by dissolving 0.6110 g of the tris(hydroxymethyl)aminomethane in 498.0 mL of deionized water, and the pH value was adjusted to 7.55 by adding a small amount of 3 M HCl. The ca. 10 mM tryptophan solution was prepared by dissolving ca. 21.6 mg of l-tryptophan in 10 mL of the aforementioned tris base buffer. The HSA and BSA aqueous samples were prepared by dissolving 0.1198 g of HSA powder into 3.0 mL of tris buffer and dissolving 0.1202 g of BSA powder into 3.0 mL of tris buffer, respectively. The concentrations of HSA and BSA were ca. 40 mg mL^–1^, similar to physiological concentrations.^35^

### Steady-State Ultraviolet (UV) Absorption and Fluorescence Spectrometers

A spectrometer (USB4000-UV–vis, Ocean Optics, 200–850 nm) with a deuterium lamp (SLS204, Thorlabs, 200–700 nm) was used to acquire the absorption spectra of the aforementioned samples. A quartz cuvette with an optical length of 100 μm (106–0.10–40, Hellma Analytics) was used to load the samples. Another spectrometer (F-7000, Hitachi) with a thermostat (qpod and TC 125, Quantum Northwest) in the sample compartment was used to record the fluorescence spectra at temperatures of 25–45 °C upon excitation at 300 nm. The dissolved tryptophan, HSA, and BSA were loaded into a 2 mm × 10 mm cuvette (104002F-10–40, Hellma Analytics), and their corresponding fluorescence spectra at 25–45 °C are shown in Figure S1 with the normalized spectra in the insets in the Supporting Information. The relative intensity changes with respect to the fluorescence intensity at 25 °C at 350–500 nm are shown in Figure S2. The normalized fluorescence spectra of dissolved tryptophan, BSA, and HSA at 30, 35, and 40 °C are presented in Figure S3.

### T-Jump Instrumentation

**Figure 1:**
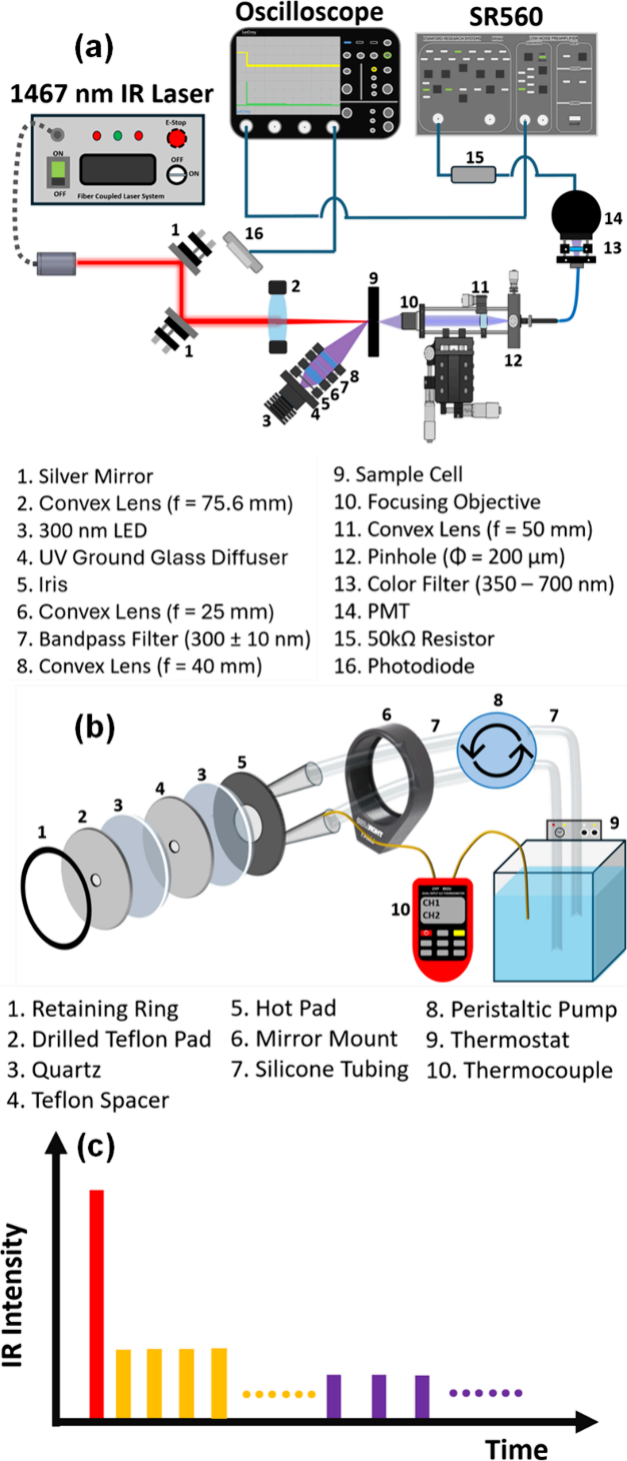
(a) Experimental setup; (b) assembly of the sample cell; (c) schematic interpretation of the infrared pulse sequence for optical heating of aqueous sample.

The apparatus was constructed based on a previous design^30,31^ using an intensity-modulated infrared diode laser, as shown in Figure 1a. A pulsed 1467 nm diode laser (FC-1450 nm-10W-ED42771, CNI Laser) operated at a repetition rate of 1 kHz was directed by silver mirrors and focused to a diameter of 1.1 mm by a biconvex lens (f = 75.6 mm, KBX058, Newport) onto the center of the sample cell (Figure 1b) to excite H_2_O for rapid temperature increase and a high temperature plateau by tuning the intensity sequences, as schematically illustrated in Figure 1c. The first pulse at full power, as indicated with the red bar, created an instantaneous temperature increase. The following lower-intensity pulses, as indicated with yellow and violet bars, were used to compensate the thermalization after the quick heating and to retain the high temperature plateau, respectively.

The assembly of the sample cell is illustrated in Figure 1b. The aqueous samples, ca. 1 μL, were sandwiched by two quartz windows (thickness: 2 mm, diameter: 25.0 mm, #14–962, Edmund Optics) separated by a Teflon spacer with a thickness of 0.8 mm and a centrally drilled hole with a diameter of 1.2 mm. A homemade thermostatic hot pad was assembled by sandwiching two 1-in. center-drilled aluminum plates with a 5 mm circular hole, which were separated by two gaskets of outer diameters ca. 25 and 5 mm with a thickness of 1 mm. One of the aluminum plates was drilled with two holes for connecting to the peristaltic pump (MP-1000, EYELA) to circulate the warm water from the external thermostat (RTE-210, Endocal) in the cavity of this hot pad.^31^ The thermostatic hot pad was attached to the sample cell to maintain the stationary temperature before T-jump. Another center-drilled Teflon pad was used to sandwich the sample cell at the opposite side of the hot pad to prevent thermalization as much as possible. A K/J-type thermocouple (CHY-802U, CHY) was inserted into the return pipe from the hot pad to measure the stationary temperature. The sample cell and the hot pad were assembled in the lens mount (LMR1/M, Thorlabs) and placed in the detection compartment. Generally, this module was thermalized for 5 min before the T-jump experiments were performed.

A 300 nm light source was used to excite dissolved tryptophan and the constituent tryptophans in HSA and BSA without exciting the constituent tyrosines. The 300 nm ultraviolet light from an LED lamp (M300L4, Thorlabs) was homogenized after passing through a diffuser (DGUV10–1500, Thorlabs) and an iris (SM1D12, Thorlabs) and further focused by a pair of convex lenses (f = 25.0 mm, #48–304, Edmund Optics; f = 40.0 mm, LA4306-UV, Thorlabs) onto the sample cell from the same side as the infrared light entrance at an angle of ca. 45°. Another bandpass filter (300 ± 10 nm, #35–884, Edmund Optics) was placed between the two lenses to better define the excitation wavelength. The fluorescence from the sample upon 300 nm excitation was collected with an objective (LMU-20X-NUV, Thorlabs, 325–500 nm) on the opposite side of the entrance of the infrared laser and later focused through a 200 μm pinhole (P200D, Thorlabs) using a lens (LA4148-UV, Thorlabs) to achieve the spatial-resolution capability. Then the fluorescence was guided to a photomultiplier tube (R928, Hamamatsu) via an optical fiber (QP400–025-SR, Ocean Optics), and a color filter (KG-3–25.4, Lambda Research Optics, 350–700 nm) was mounted in front of the photomultiplier tube to better define the detection wavelengths in the 350–500 nm range. The voltage signal from the photomultiplier tube was transmitted through a 50 kΩ resistor and electronically filtered at <1k Hz by a voltage preamplifier (SR560, Stanford Research Systems) before being recorded with an oscilloscope (WaveSurfer 24MXs-B, LeCroy).

## Results and Discussion

The fluorescence intensity of tryptophan is sensitive to temperature^36^ and has a temperature dependence of ca. −2% °C^–1^.^37^ Thus, the fluorescence intensity evolution of tryptophan served as the fluorescent thermometer in the heating volume. To validate the performance of this apparatus, the protein dynamics of HSA and BSA were examined. Although HSA and BSA have great similarity in their sequences and structures,^38,39^ HSA has only one tryptophan at the 214th residue,^40^ while BSA has two tryptophans at the 134th and 213th residues.^41^ Trp-214 of HSA and Trp-213 of BSA both stay in their hydrophobic folds, whereas Trp-134 of BSA stays at the surface.^42,43^ In addition, irreversible denaturing of HSA and BSA occurs at 55 and 50 °C, respectively.^44,45^ Therefore, the following T-jump experiments were performed by instantaneous heating of HSA and BSA solution to 36.6–39.9 °C from the initial temperature *T*_0_ = 35.0 °C, which was still within the physiological temperature range, and the dynamic process was attributed to the reversible thermally induced structural alteration.

### Tunability of Temperature Increment and Plateaued Temperature

**Figure 2:**
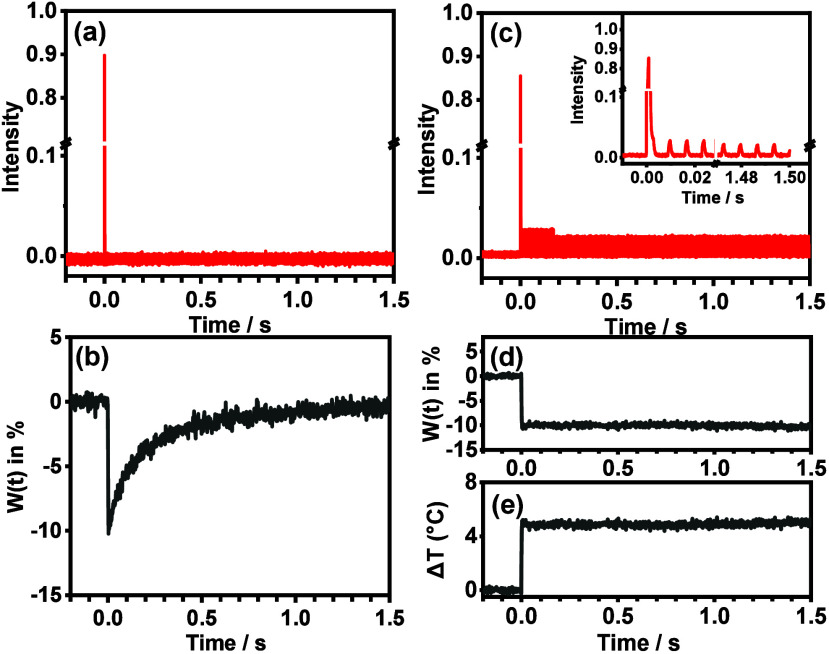
(a) The intensity evolution of the single-shot infrared pulse for excitation of dissolved tryptophan; (b) the relative fluorescence intensity change evolutions of dissolved tryptophan, *W*(*t*) = Δ*I*(*t*)/*I*_0_, upon excitation in (a); (c) the intensity evolution for the “optical Riemann sum” of the infrared pulses for excitation of dissolved tryptophan, with the inset for the periods of −0.01 to 0.028 and 1.47 to 1.50 s; (d) the resultant *W*(*t*), and (e) the corresponding temperature change evolutions (Δ*T*(*t*)) derived using eq 1 upon the excitation in (c). The temporal profile in (d) was averaged from 15 identical operations.

Upon excitation of dissolved tryptophan solution by a single full-powered infrared pulse, as presented in Figure 2a, the evolution of its relative fluorescence change (*W*(*t*) = Δ*I*(*t*)/*I*_0_), as shown in Figure 2b, manifested a quick decrease in ca. 2 ms and a gradual recovery due to the thermalization, which hampered the characterization of the protein dynamics within several tens of milliseconds at the jumped temperature. Thus, a conceptual “optical Riemann sum” heating process was used to generate an instantaneous temperature jump and a high temperature plateau by changing the intensities of a series of IR pulse sequences. When one single pulse of ca. 80% of the full power was applied first, followed by 25 pulses of 11% of the full power at time intervals of 6 ms and cascading pulses of 10% of full power at time intervals of 6 ms for the remaining period, as indicated in Figure 2c and the inset, the evolution of *W*(*t*) manifested a quick decrease, followed by a constant intensity in the prolonged period, as shown in Figure 2d. After transformation of the *W*(*t*) with the conversion factor α = −2.06% °C^–1^ in eq 1, which was derived from Figure S2, the temperature change evolution, Δ*T*(*t*), was derived as shown in Figure 2e, manifesting a jumped temperature of ca. 5.0 °C in ca. 2 ms.
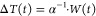
1

**Figure 3:**
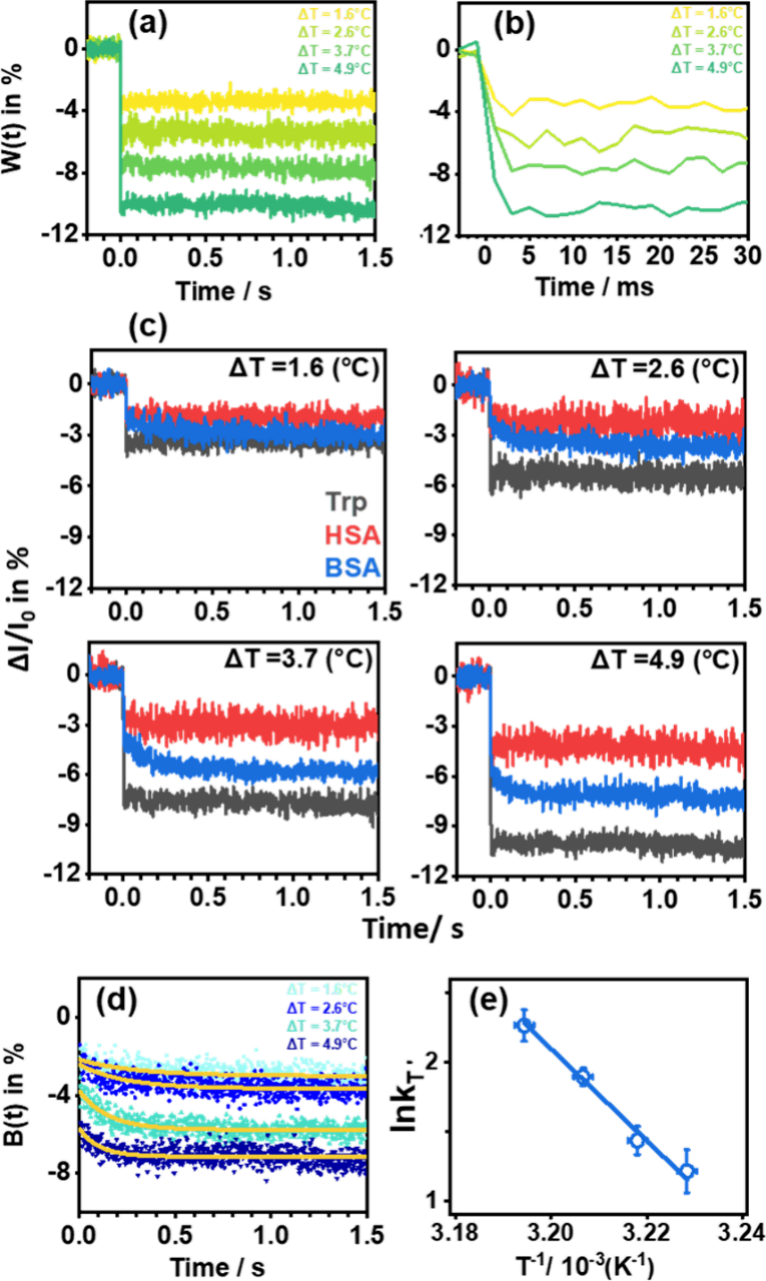
(a) *W*(*t*) of dissolved tryptophan upon heating by 1.6, 2.6, 3.7, and 4.9 °C from 35.0 °C; (b) the enlarged view of (a) at 30 ms; (c) the comparison of *W*(*t*), *H*(*t*), and *B*(*t*) upon heating using the conditions in (a); (d) the comparison of the observed temporal profiles of *B*(*t*) in dots and the fitted profiles with eq 8 in orange solid lines; (e) the plot of ln *k*_*T*′_ versus  to derive the apparent activation energy *E*_a_.

The plateaued steady-state temperature increase (Δ*T*_ss_ in °C) was derived from the plateaued stationary intensity drop (γ in %) in the prolonged excitation period using eq 2,
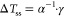
2Δ*T*_ss_ was adjusted by changing the intensity of the first pulse and slightly tuning the intensities of the following pulses without greatly perturbing the rising time (ca. 2 ms), as revealed in Figures 3a and 3b. If more intense pulses were employed in the instantaneous heating, the jumped temperature can be greatly lifted up but the rising time will be retarded. For example, by applying 3 pulses of 80% of the full power at time intervals of 1 ms, followed by 70 pulses of 14% of the full power at time intervals of 6 ms and cascading pulses of 13% of full power at time intervals of 11 ms for the remaining period (Figure S4a), the dissolved tryptophan fluorescence intensity suddenly dropped (Figure S4b) and the corresponding temperature can jump with a rising time of ca. 5 ms with a stationary high temperature of ca. 14 °C (Figures S4c and S4d**)**. Therefore, the excitation powers of the pulses should be adjusted to achieve the expected heating profile. In the present experimental configuration, a high temperature plateau lasting for 5 s can be easily achieved. It is noteworthy that the instantaneous high temperature should be carefully controlled to avoid the irreversible thermal-denaturing of the targeted proteins.

### Quantification of the Kinetics from the Evolution of the Fluorescence Intensity Change of a Given Protein upon T-Jump

Generally, an apparent reversible process was assumed to describe the transformation between two hypothetical states, **A** and **B**, initiated by temperature jump, as written in eq 3.
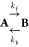
3

The corresponding kinetics was interpreted using a single exponential component with a rate coefficient *k*_*T*′_, which included the forward and backward rate coefficients, *k*_f_ and *k*_b_, respectively, at the jumped temperature (*T*′ = *T*_0_ + Δ*T*); i.e., *k*_*T*′_ = *k*_f,*T*′_ + *k*_b,*T*′_, and *k*_*T*′_ was commonly treated as an apparent relaxation kinetic rate coefficient.^22,46^ As the protein was instantaneously heated to the high temperature before the protein dynamics ensued, both state **A** and state **B** contributed the overall tryptophan fluorescence intensity at time zero and at high temperature *T*′, *I*_*T*′_(0), as expressed in eq 4,^28^
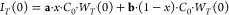
4where **a** and **b** represent the relative quantum yields of the tryptophan fluorescence in state **A** and state **B** with respect to the pure tryptophan in aqueous solution at *T*′, and *x* and (1 – *x*) denote the molar fractions in state **A** and state **B** at *T*′ before starting the protein dynamics, respectively. C_0_ denotes the total concentration of the given protein. W_*T*′_(0) denotes the fluorescence intensity of dissolved tryptophan in solution at *T*′. When the protein began the dynamic process at *T*′, the transition between state **A** and state **B** led to a fluorescence intensity change if **a** ≠ **b**. The corresponding intensity evolution at *T*′, *I*_*T*′_(t), is expressed as eq 5,
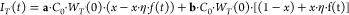
5where *f*(*t*) denotes the dynamic process using an apparent single exponential component with a rate coefficient *k*_*T*′_, as written in eq 6,
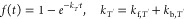
6where η represents the percentile of the protein sample pursuing the protein dynamics *f*(*t*) from state **A**. Accordingly, the relative fluorescence intensity change evolution,  can be expressed in eq 7,
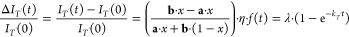
7where λ, which is equal to , denotes the apparent amplitude of the protein dynamics. The derived *k*_*T*′_ at varied *T*′ was used to derive the apparent activation energy (*E*_a_) of the dynamic process from the slope of the linear progression of ln *k*_*T*′_ versus *T*′^–1^ using Arrhenius analyses.^22,23,46,47^

### The Differences in the Protein Dynamics of HSA and BSA Revealed by Their Intrinsic Tryptophan Fluorescence Evolutions

The fluorescence spectrum of tryptophan is more bathochromic in a hydrophilic environment than in a hydrophobic environment.^48^ Therefore, the red-shifted fluorescence spectrum of BSA, compared to that of HSA in Figure S3, indicated that the tryptophans in BSA could be more water-exposed. From comparing the similarities in the sequences of HSA and BSA,^38,39^ the red-shifted fluorescent component was attributed to Trp-134 of BSA,^29^ which stayed at the surface of BSA^42,43^ and probably possessed water accessibility. Concomitantly, the relative fluorescence change evolutions of tryptophan (*W*(*t*)), HSA (*H*(*t*)), and BSA (*B*(*t*)) upon heating to different plateaued temperatures were intrinsically different, as shown in Figure 3c. *W*(*t*) manifested instantaneous drops due to the heating, followed by a stationary intensity owing to the stationary plateaued temperature. *H*(*t*) manifested temporal profiles similar to those of *W*(*t*) without a dynamic component within 1.5 s, no matter what the high temperature was. Accordingly, HSA only manifested the instantaneous temperature response without the dynamic component in the prolonged period, probably suggesting that no significant structural alteration occurred at the vicinity of Trp-214 of HSA upon thermal stimulus. Alternatively, if the hypothetical states **A** and **B** had possessed the same relative quantum yields **a** and **b**, the fluorescence intensity change would have vanished, based on eq 7, and the protein dynamics would not have been revealed.

However, *B*(*t*) manifested a quick intensity drop in response to the instantaneous temperature increase and an extra dynamic component in the prolonged period, which became accelerated and intensified as the jumped temperature increased. Since HSA and BSA possess great similarity in their sequences and Trp-214 of HSA and Trp-213 of BSA both stay in their hydrophobic folds, the observed protein dynamics in BSA were probably attributed to the structural alteration at Trp-134 of BSA, which stays at the surface.^42,43^ To quantify the kinetics of dynamic process *f*(*t*) embedded in *B*(*t*) at varied *T*′, one apparent exponential component was used to fit the temporal profiles of *B*(*t*) in Figure 3c using eq 8, which was modified from eq 7,
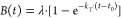
8where *t*_0_ was fixed at 3 ms to account for the time zero of the corresponding dynamic process starting at *T*′, and the resultant profiles are shown in Figure 3d. The values of *k*_*T*′_ are summarized in Table 1. A linear regression was used to fit ln *k*_*T*′_ versus  in Figure 3e and the slope, i.e. *E*_a_, was determined to be 276 ± 23 kJ mol^–1^. This value can be treated as an intrinsic property of the native BSA. When the drugs or the ligands were conjugated with BSA, the change in *E*_a_ would reveal the extent of the effects of these extra chemicals. At present, however, it would be difficult or even unrealistic to identify the origins of this energy barrier because it involves the mutual interactions of the residue network, the fission and reformation of hydrogen bonding, the solvent accessibility, etc.

**Table 1:** Relevant Parameters Derived from *W*(*t*) and *B*(*t*)^a^

Δ*T*_ss_ (°C)	1.6 ± 0.2	2.6 ± 0.2	3.7 ± 0.2	4.9 ± 0.2
*T*′ (°C)	36.6 ± 0.2	37.6 ± 0.2	38.7 ± 0.2	39.9 ± 0.2
γ^b^	–3.38 ± 0.32	–5.43 ± 0.47	–7.64 ± 0.37	–10.07 ± 0.35
*k*_*T*′_ (s^–1^)	3.36 ± 0.52	4.20 ± 0.43	6.66 ± 0.41	9.65 ± 1.11

a The initial temperature was controlled at 35.0 ± 0.1 °C.

b Derived from eq 2.

## Conclusions and Bioanalytical Implications

In this work, we have constructed a fluorescence-based T-jump apparatus with a temporal resolution of ca. 2 ms. The sequential infrared pulses at different intensities were programmed to tune the instantaneous increase in the temperature. Generally, a tiny amount of sample, ca. 1 μL, was required for the measurement, and the data acquisition of  for a given sample and one jumped temperature upon 15 averages could be readily finished within 3 h. This apparatus was validated by differentiating the protein dynamics of the model proteins, BSA and HSA. The fluorescence intensity change evolutions of HSA and dissolved tryptophan upon T-jump were similar, implying a sole thermal response without a dynamic process. In contrast, the fluorescence intensity change evolution of BSA possessed a quick drop, owing to the temperature increase, and a slow dynamic process, whose activation energy was quantified as 276 ± 23 kJ mol^–1^.

Complementary to the conventional spectroscopic methods, such as circular dichroism and infrared, used to characterize the steady-state secondary structures of a given protein, the presented apparatus provided the dynamic information on the targeted protein upon thermal stimulation. In addition, the millisecond temporal resolution provided the capability to unveil the collective large-domain motions of the protein, supplementing the localized structural alteration on the time scale of nanoseconds to microseconds studied with the fast T-jump methods using pulsed infrared laser.

## Supporting Information Available

The steady-state fluorescence spectra of dissolved tryptophan, HSA, and BSA at temperatures of 25–45 °C (**Figure S1**), relative integrated fluorescence intensity difference of dissolved tryptophan, HSA, and BSA (**Figure S2**), the normalized fluorescence spectra of tryptophan, HSA, and BSA (**Figure S3**), and another heating scenario (**Figure S4**) (PDF)

## References

[ref1] Nam K.; Wolf-Watz M. Protein Dynamics: The Future Is Bright and Complicated!. Struct. Dyn. 2023, 10, 014301 10.1063/4.0000179. 36865927 PMC9974214

[ref2] Kovermann M.; Rogne P.; Wolf-Watz M. Protein Dynamics and Function from Solution State NMR Spectroscopy. Q. Rev. Biophys. 2016, 49, e6 10.1017/S0033583516000019. 27088887

[ref3] Frauenfelder H.; Sligar S. G.; Wolynes P. G. The Energy Landscapes and Motions of Proteins. Science 1991, 254, 1598–1603. 10.1126/science.1749933. 1749933

[ref4] Henzler-Wildman K.; Kern D. Dynamic Personalities of Proteins. Nature 2007, 450, 964–972. 10.1038/nature06522. 18075575

[ref5] Li C.; Tang C.; Liu M. Protein Dynamics Elucidated by NMR Technique. Protein Cell 2013, 4, 726–730. 10.1007/s13238-013-3912-1. 24104391 PMC4875435

[ref6] Hu Y.; Cheng K.; He L.; Zhang X.; Jiang B.; Jiang L.; Li C.; Wang G.; Yang Y.; Liu M. NMR-Based Methods for Protein Analysis. Anal. Chem. 2021, 93, 1866–1879. 10.1021/acs.analchem.0c03830. 33439619

[ref7] Lorenz U. J. Microsecond Time-resolved Cryo-electron Microscopy. Curr. Opin. Struct. Biol. 2024, 87, 102840 10.1016/j.sbi.2024.102840. 38810313

[ref8] Amann S. J.; Keihsler D.; Bodrug T.; Brown N. G.; Haselbach D. Frozen in Time: Analyzing Molecular Dynamics with Time-resolved Cryo-EM. Structure 2023, 31, 4–19. 10.1016/j.str.2022.11.014. 36584678 PMC9825670

[ref9] Kawale A. A.; Burmann B. M. Characterization of Backbone Dynamics Using Solution NMR Spectroscopy to Discern the Functional Plasticity of Structurally Analogous Proteins. STAR Protoc. 2021, 2, 100919 10.1016/j.xpro.2021.100919. 34761231 PMC8567434

[ref10] Phillips C. M.; Mizutani Y.; Hochstrasser R. M. Ultrafast Thermally Induced Unfolding of RNase A. Proc. Natl. Acad. Sci. U. S. A. 1995, 92, 7292–7296. 10.1073/pnas.92.16.7292. 7638183 PMC41325

[ref11] Dyer R. B.; Gai F.; Woodruff W. H.; Gilmanshin R.; Callender R. H. Infrared Studies of Fast Events in Protein Folding. Acc. Chem. Res. 1998, 31, 709–716. 10.1021/ar970343a.

[ref12] Jemth P.; Gianni S.; Day R.; Li B.; Johnson C. M.; Daggett V.; Fersht A. R. Demonstration of a Low-energy on-pathway Intermediate in a Fast-folding Protein by Kinetics, Protein Engineering, and Simulation. Proc. Natl. Acad. Sci. U. S. A. 2004, 101, 6450–6455. 10.1073/pnas.0401732101. 15096617 PMC404065

[ref13] Huang C.-Y.; Balakrishnan G.; Spiro T. G. Early Events in Apomyoglobin Unfolding Probed by Laser T-jump/UV Resonance Raman Spectroscopy. Biochemistry 2005, 44, 15734–15742. 10.1021/bi051578u. 16313176

[ref14] Chung H. S.; Khalil M.; Smith A. W.; Tokmakoff A. Transient Two-dimensional IR Spectrometer for Probing Nanosecond Temperature-jump Kinetics. Rev. Sci. Instrum. 2007, 78, 063101 10.1063/1.2743168. 17614599

[ref15] Hart T.; Hosszu L. L. P.; Trevitt C. R.; Jackson G. S.; Waltho J. P.; Collinge J.; Clarke A. R. Folding Kinetics of the Human Prion Protein Probed by Temperature Jump. Proc. Natl. Acad. Sci. U. S. A. 2009, 106, 5651–5656. 10.1073/pnas.0811457106. 19321423 PMC2667024

[ref16] Kubelka J. Time-resolved Methods in Biophysics. 9. Laser Temperature-Jump Methods for Investigating Biomolecular Dynamics. Photochem. Photobiol. Sci. 2009, 8, 499–512. 10.1039/b819929a. 19337664

[ref17] Ebbinghaus S.; Dhar A.; McDonald J. D.; Gruebele M. Protein Folding Stability and Dynamics Imaged in a Living Cell. Nat. Methods 2010, 7, 319–323. 10.1038/nmeth.1435. 20190760

[ref18] Khuc M.-T.; Mendonça L.; Sharma S.; Solinas X.; Volk M.; Hache F. Measurement of Circular Dichroism Dynamics in a Nanosecond Temperature-Jump Experiment. Rev. Sci. Instrum. 2011, 82, 054302 10.1063/1.3592331. 21639524

[ref19] Jones K. C.; Peng C. S.; Tokmakoff A. Folding of a Heterogeneous β-hairpin Peptide from Temperature-jump 2D IR Spectroscopy. Proc. Natl. Acad. Sci. U. S. A. 2013, 110, 2828–2833. 10.1073/pnas.1211968110. 23382249 PMC3581973

[ref20] Wirth A. J.; Liu Y.; Prigozhin M. B.; Schulten K.; Gruebele M. Comparing Fast Pressure Jump and Temperature Jump Protein Folding Experiments and Simulations. J. Am. Chem. Soc. 2015, 137, 7152–7159. 10.1021/jacs.5b02474. 25988868 PMC4794261

[ref21] Meadows C. W.; Balakrishnan G.; Kier B. L.; Spiro T. G.; Klinman J. P. Temperature-Jump Fluorescence Provides Evidence for Fully Reversible Microsecond Dynamics in a Thermophilic Alcohol Dehydrogenase. J. Am. Chem. Soc. 2015, 137, 10060–10063. 10.1021/jacs.5b04413. 26223665 PMC4970856

[ref22] Ding B.; Hilaire M. R.; Gai F. Infrared and Fluorescence Assessment of Protein Dynamics: From Folding to Function. J. Phys. Chem. B 2016, 120, 5103–5113. 10.1021/acs.jpcb.6b03199. 27183318 PMC4911233

[ref23] Davis C. M.; Dyer R. B. The Role of Electrostatic Interactions in Folding of β-Proteins. J. Am. Chem. Soc. 2016, 138, 1456–1464. 10.1021/jacs.5b13201. 26750867 PMC4749129

[ref24] Toto A.; Bonetti D.; De Simone A.; Gianni S. Understanding the Mechanism of Binding between Gab2 and the C Terminal SH3 Domain from Grb2. Oncotarget 2017, 8, 82344–82351. 10.18632/oncotarget.19323. 29137268 PMC5669894

[ref25] Chen K.-J.; Lin C.-T.; Tseng K.-C.; Chu L.-K. Using SiO_2_-Coated Gold Nanorods as Temperature Jump Photothermal Convertors Coupled with a Confocal Fluorescent Thermometer to Study Protein Unfolding Kinetics: A Case of Bovine Serum Albumin. J. Phys. Chem. C 2017, 121, 14981–14989. 10.1021/acs.jpcc.7b05033.

[ref26] Menssen R. J.; Tokmakoff A. Length-Dependent Melting Kinetics of Short DNA Oligonucleotides Using Temperature-Jump IR Spectroscopy. J. Phys. Chem. B 2019, 123, 756–767. 10.1021/acs.jpcb.8b09487. 30614693

[ref27] Ashwood B.; Lewis N. H. C.; Sanstead P. J.; Tokmakoff A. Temperature-Jump 2D IR Spectroscopy with Intensity-Modulated CW Optical Heating. J. Phys. Chem. B 2020, 124, 8665–8677. 10.1021/acs.jpcb.0c07177. 32902979 PMC7850621

[ref28] Li H.-Y.; Tseng K.-C.; Chu L.-K. Tier-0 Protein Dynamics of Bovine Serum Albumin: A Kinetics and Energetics Study of the Collective Domain Motions. Chem. Phys. Lett. 2021, 762, 138102 10.1016/j.cplett.2020.138102.

[ref29] Wang P.-Y.; Yang C.-T.; Chu L.-K. Differentiating the Protein Dynamics Using Fluorescence Evolution of Tryptophan Residue(s): A Comparative Study of Bovine and Human Serum Albumins upon Temperature Jump. Chem. Phys. Lett. 2021, 781, 138998 10.1016/j.cplett.2021.138998.

[ref30] Yang C.-T.; Chu L.-K. Protein Dynamics of Human Serum Albumin at Hypothermic Temperatures Investigated by Temperature Jump. Phys. Chem. Chem. Phys. 2022, 24, 11079–11085. 10.1039/D2CP00220E. 35471209

[ref31] Kao T.-L.; Chu L.-K. Alcohol-induced Retarded Protein Dynamics of Human Serum Albumin Unveiled by Temperature Jump. Chem. Phys. Lett. 2023, 833, 140899 10.1016/j.cplett.2023.140899.

[ref32] Reich R. M.; Sutter J. R. Capacitor Discharge Temperature-jump Apparatus with Nanosecond Capabilities. Anal. Chem. 1977, 49, 1081–1085. 10.1021/ac50015a054.

[ref33] Ameen S.; De Maeyer L. Stimulated Raman Scattering from H_2_ Gas for Fast Heating in Laser-Temperature-Jump Chemical Relaxation Experiments. J. Am. Chem. Soc. 1975, 97, 1590–1591. 10.1021/ja00839a061.

[ref34] Williams A. P.; Longfellow C. E.; Freier S. M.; Kierzek R.; Turner D. H. Laser Temperature-Jump, Spectroscopic, and Thermodynamic Study of Salt Effects on Duplex Formation by dGCATGC. Biochemistry 1989, 28, 4283–4291. 10.1021/bi00436a025. 2765487

[ref35] Carter D. C.; Ho J. X. Structure of Serum Albumin. Adv. Protein Chem. 1994, 45, 153–203. 10.1016/S0065-3233(08)60640-3. 8154369

[ref36] Gally J. A.; Edelman G. M. The Effect of Temperature on the Fluorescence of Some Aromatic Amino Acids and Proteins. Biochim. Biophys. Acta 1962, 60, 499–509. 10.1016/0006-3002(62)90869-7. 13896484

[ref37] Chiu M.-J.; Chu L.-K. Quantifying the Photothermal Efficiency of Gold Nanoparticles Using Tryptophan as an *in situ* Fluorescent Thermometer. Phys. Chem. Chem. Phys. 2015, 17, 17090–17100. 10.1039/C5CP02620B. 26068797

[ref38] Huang B. X.; Kim H.-Y.; Dass C. Probing Three-dimensional Structure of Bovine Serum Albumin by Chemical Cross-linking and Mass Spectrometry. J. Am. Soc. Mass Spectrom. 2004, 15, 1237–1247. 10.1016/j.jasms.2004.05.004. 15276171

[ref39] Majorek K. A.; Porebski P. J.; Dayal A.; Zimmerman M. D.; Jablonska K.; Stewart A. J.; Chruszcz M.; Minor W. Structural and Immunologic Characterization of Bovine, Horse, and Rabbit Serum Albumins. Mol. Immunol. 2012, 52, 174–182. 10.1016/j.molimm.2012.05.011. 22677715 PMC3401331

[ref40] Minghetti P. P.; Ruffner D. E.; Kuang W.-J.; Dennison O. E.; Hawkins J. W.; Beattie W. G.; Dugaiczyk A. Molecular Structure of the Human Albumin Gene Is Revealed by Nucleotide Sequence within q11–22 of Chromosome 4. J. Biol. Chem. 1986, 261, 6747–6757. 10.1016/S0021-9258(19)62680-3. 3009475

[ref41] Hirayama K.; Akashi S.; Furuya M.; Fukuhara K.-i. Rapid Confirmation and Revision of the Primary Structure of Bovine Serum Albumin by ESIMS and Frit-FAB LC/MS. Biochem. Biophys. Res. Commun. 1990, 173, 639–646. 10.1016/S0006-291X(05)80083-X. 2260975

[ref42] Peterman B. F.; Laidler K. J. Study of Reactivity of Tryptophan Residues in Serum Albumins and Lysozyme by N-bromosuccinamide Fluorescence Quenching. Arch. Biochem. Biophys. 1980, 199, 158–164. 10.1016/0003-9861(80)90268-4. 7188848

[ref43] Punyiczki M.; Rosenberg A. The Effect of Viscosity on the Accessibility of the Single Tryptophan in Human Serum Albumin. Biophys. Chem. 1992, 42, 93–100. 10.1016/0301-4622(92)80011-S. 1581518

[ref44] Rezaei-Tavirani M.; Moghaddamnia S. H.; Ranjbar B.; Amani M.; Marashi S.-A. Conformational Study of Human Serum Albumin in Pre-denaturation Temperatures by Differential Scanning Calorimetry, Circular Dichroism and UV Spectroscopy. J. Biochem. Mol. Biol. 2006, 39, 530–536. 10.5483/BMBRep.2006.39.5.530. 17002873

[ref45] Lin V. J. C.; Koenig J. K. Raman Studies of Bovine Serum Albumin. Biopolymers 1976, 15, 203–218. 10.1002/bip.1976.360150114. 1119

[ref46] Vaughn M. B.; Zhang J.; Spiro T. G.; Dyer R. B.; Klinman J. P. Activity-Related Microsecond Dynamics Revealed by Temperature-Jump Förster Resonance Energy Transfer Measurements on Thermophilic Alcohol Dehydrogenase. J. Am. Chem. Soc. 2018, 140, 900–903. 10.1021/jacs.7b12369. 29323490 PMC5802873

[ref47] Vu D. M.; Brewer S. H.; Dyer R. B. Early Turn Formation and Chain Collapse Drive Fast Folding of the Major Cold Shock Protein CspA of Escherichia coli. Biochemistry 2012, 51, 9104–9111. 10.1021/bi301296y. 23098216 PMC3567219

[ref48] Reshetnyak Y. K.; Burstein E. A. Decomposition of Protein Tryptophan Fluorescence Spectra into Log-Normal Components. II. The Statistical Proof of Discreteness of Tryptophan Classes in Proteins. Biophys. J. 2001, 81, 1710–1734. 10.1016/S0006-3495(01)75824-9. 11509383 PMC1301648

