# Supplementary material for: A Fluorescence-Based Temperature-Jump Apparatus for Illustrating Protein Dynamics on the Millisecond Time Scale
Source: Anal Chem. 2025 Feb 13;97(7):3810–5. doi: 10.1021/acs.analchem.4c06501 (PMC11866288; doi:10.1021/acs.analchem.4c06501)
Supplement: Supplementary file 1 — ac4c06501_si_001.pdf [file ac4c06501_si_001.pdf]

Supporting Information

A Fluorescence-Based Temperature-Jump Apparatus for Illustrating Protein Dynamics on the Millisecond Time Scale

*Liang-Che Kung and Li-Kang Chu**

Department of Chemistry, National Tsing Hua University, 101, Sec. 2, Kuang-Fu Road, Hsinchu 300044, Taiwan

Corresponding Author

*To whom correspondence should be addressed. Phone: 886-3-5715131 ext. 33396. Fax: 886-3-5711082. E-mail: lkchu@mx.nthu.edu.tw.

Table of contents

Figure S1. Fluorescence spectra of dissolved tryptophan, HSA, and BSA in aqueous solution at 25–45 °C upon 300 nm excitation.

Figure S2. Relative integrated fluorescence intensity difference of dissolved tryptophan, HSA, and BSA at 350–500 nm upon excitation at 300 nm.

Figure S3. The normalized fluorescence spectra of dissolved tryptophan, HSA, and BSA at 30, 35, and 40 °C.

Figure S4. Another heating scenario and the corresponding temperature change evolution.

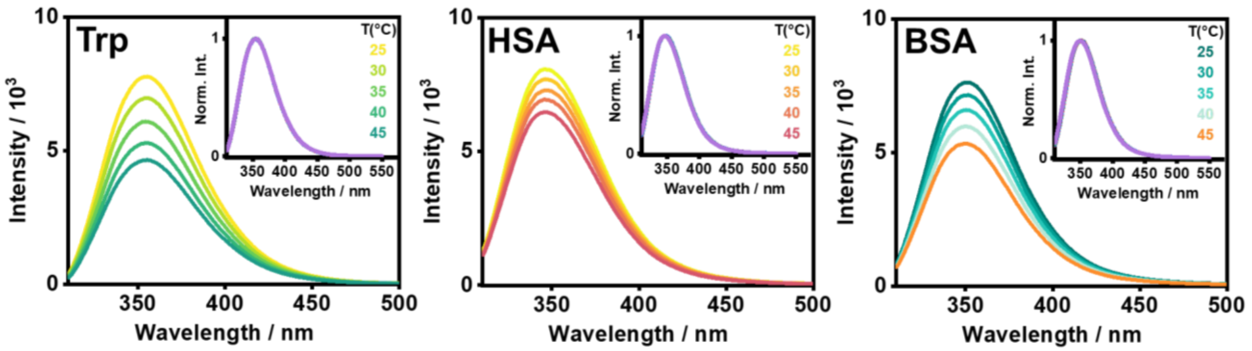

Figure S1. Fluorescence spectra of dissolved tryptophan (10 mM), HSA (570 μM), and BSA (565 μM) in aqueous solution at 25–45 °C upon 300 nm excitation. The normalized spectra are shown in the insets. The concentration of tris base buffer was 10 mM.

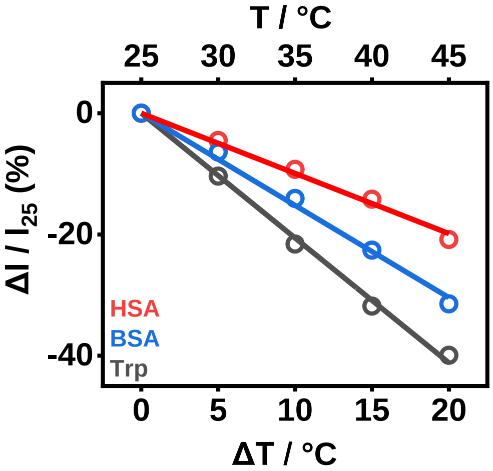

Figure S2. Relative integrated fluorescence intensity differences of dissolved tryptophan (black), HSA (red), and BSA (blue) at 350–500 nm upon excitation at 300 nm of samples in **Figure S1**. I_{25} and ΔI refer to the fluorescence intensity at 25 °C and the difference at a given temperature with respect to that at 25 °C, respectively. The ratios of $\Delta I/I_{25}$ with respect to ΔT were fitted with linear regressions.

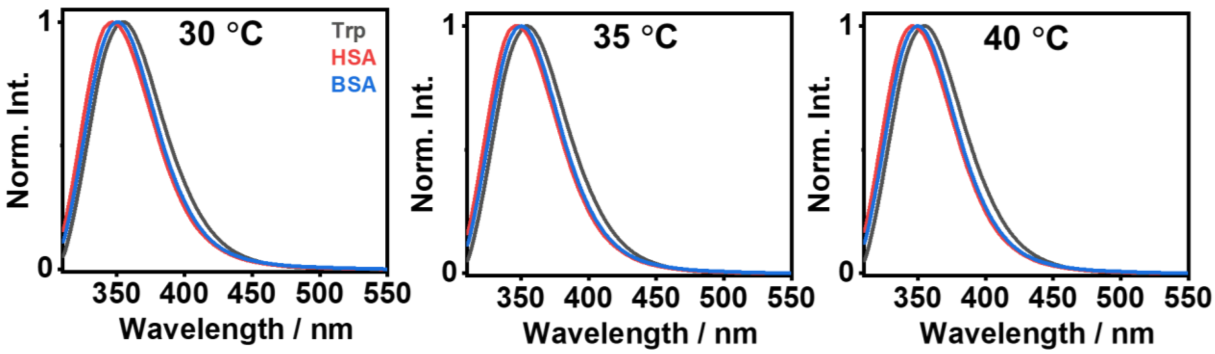

Figure S3. The normalized fluorescence spectra of dissolved tryptophan, HSA, and BSA at 30, 35, and 40 °C from **Figure S1**.

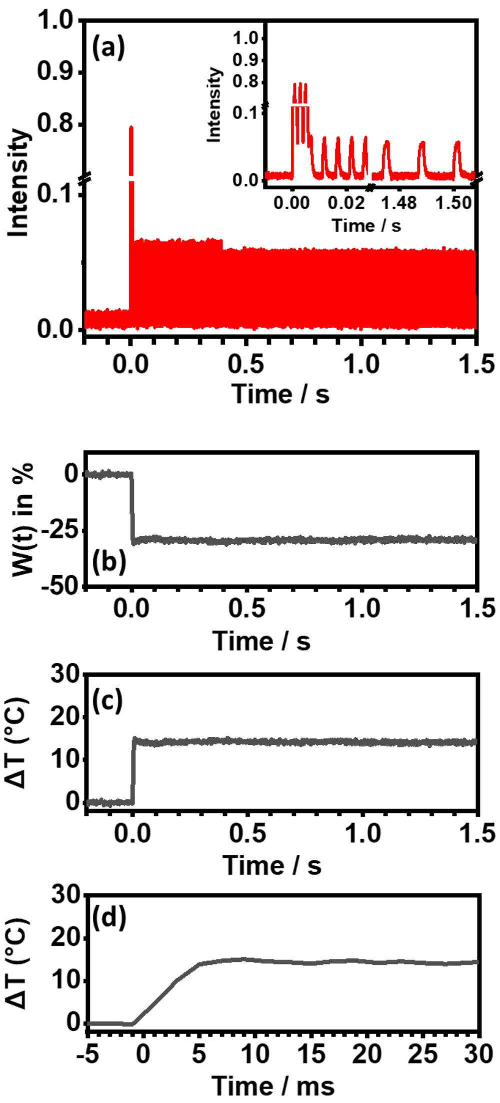

Figure S4. (a) The intensity evolution for the "optical Riemann sum" of the infrared pulses for excitation of dissolved tryptophan, with the inset for the periods of –0.01 to 0.028 and 1.47 to 1.51 s: 3 pulses of 80 % of the full power at time intervals of 1 ms, followed by 70 pulses of 14 % of the full power at time intervals of 6 ms and cascading pulses of 13 % of full power at time intervals of 10 ms for the remaining period; (b) the resultant W(t); (c) the corresponding temperature change evolutions (ΔT(t)); (d) the enlarged view of (c) at –5 to 30 ms.
